# Personalized Metabolomics for Predicting Glucose Tolerance Changes in Sedentary Women After High-Intensity Interval Training

**DOI:** 10.1038/srep06166

**Published:** 2014-08-28

**Authors:** Naomi L. Kuehnbaum, Jenna B. Gillen, Martin J. Gibala, Philip Britz-McKibbin

**Affiliations:** 1 Department of Chemistry and Chemical Biology, McMaster University, Hamilton, Canada; 2 Department of Kinesiology, McMaster University, Hamilton, Canada

## Abstract

High-intensity interval training (HIIT) offers a practical approach for enhancing cardiorespiratory fitness, however its role in improving glucose regulation among sedentary yet normoglycemic women remains unclear. Herein, multi-segment injection capillary electrophoresis-mass spectrometry is used as a high-throughput platform in metabolomics to assess dynamic responses of overweight/obese women (*BMI* > 25, *n* = 11) to standardized oral glucose tolerance tests (OGTTs) performed before and after a 6-week HIIT intervention. Various statistical methods were used to classify plasma metabolic signatures associated with post-prandial glucose and/or training status when using a repeated measures/cross-over study design. Branched-chain/aromatic amino acids and other intermediates of urea cycle and carnitine metabolism decreased over time in plasma after oral glucose loading. Adaptive exercise-induced changes to plasma thiol redox and orthinine status were measured for trained subjects while at rest in a fasting state. A multi-linear regression model was developed to predict changes in glucose tolerance based on a panel of plasma metabolites measured for naïve subjects in their untrained state. Since treatment outcomes to physical activity are variable between-subjects, prognostic markers offer a novel approach to screen for potential negative responders while designing lifestyle modifications that maximize the salutary benefits of exercise for diabetes prevention on an individual level.

Physical inactivity is a modifiable risk factor associated with an alarming increase in obesity and chronic metabolic disorders worldwide, including type 2 diabetes^1^. Regular physical activity also improves mental health and cognitive function for healthy aging^2^. Thus, exercise represents a cost-effective lifestyle intervention for disease prevention while reducing the socioeconomic burden of late-stage treatment of diabetes complications. For instance, a 10 year follow-up of intensive lifestyle modifications consisting of moderate sub-maximal exercise training lowered the incidence of diabetes by 34% in high risk adults that was about two-fold more effective than metformin therapy^3^. Sustained long-term benefits have also been shown to reduce diabetes progression over 13 years, where greater risk reduction was associated with adherence to lifestyle changes during the intervention period^4^. However, lack of time remains a major barrier of exercise training to population health that satisfies recommended minimal guidelines with less than 10% of children reported to be involved in 60 min of moderate intensity exercise daily^5^.

High-intensity interval training (HIIT) offers a practical exercise protocol that is characterized by short yet intense bursts of activity interspersed with periods of rest or low-intensity exercise. HIIT is equivalent and in many cases superior to sub-maximal continuous training while requiring less time commitments and lower total exercise volume^6^. The type of exercise, number and duration of intervals, and optimum intensity level can be adapted to meet the desired therapeutic goals and/or target populations. In adults with coronary artery disease, metabolic syndrome, and obesity, HIIT enhances cardiorespiratory fitness and other measures of cardiovascular health, such as resting blood pressure, endothelial function, and left ventricular morphology^7^. Extremely short duration HIIT protocols have also been used to improve insulin sensitivity in sedentary populations^8^, whereas two-week HIIT interventions reduce hyperglycemia^9^ and post-prandial glucose responses of patients with type 2 diabetes^10^. Although HIIT can reduce abdominal adiposity in young women^11^, the insulin-sensitizing benefits associated with exercise training are independent from weight loss or body fat reduction^12^. Moreover, adaptive physiological changes to HIIT remain poorly understood with treatment responses variable between-subjects^13^. Exercise training can also elicit unanticipated adverse outcomes in susceptible individuals that are difficult to predict based on body mass or composition^14^.

Metabolomics offers a systemic approach for elucidating complex mechanisms associated with nutritional and/or exercise interventions relevant to human health^15^. Challenge tests in particular provide deeper insight into changes in human metabolism that is often not captured from baseline/single-point measurements alone^16^. For instance, oral glucose tolerance tests (OGTTs) evaluate subtle perturbations in metabolism and diabetes risk based on dynamic responses after high-dose oral glucose loading^17^,^18^,^19^. Glucose, insulin, and glycated haemoglobin A1c are widely used indicators of glucose homeostasis for the diagnosis or treatment monitoring of diabetes; however, other classes of metabolites are implicated in impaired glucose tolerance as related to four major axes of insulin action, including proteolysis, lipolysis, ketogenesis and glycolysis^20^. To the best of our knowledge, this is the first metabolomics study to examine adaptive changes in glucose tolerance after exercise training in women without impaired insulin sensitivity^21^. A cohort of overweight/obese women were recruited to participate in a 6-week HIIT intervention, and standardized OGTTs were performed for each subject prior to and after exercise training using a repeated measures/cross-over study design. Multi-segment injection-capillary electrophoresis-mass spectrometry (MSI-CE-MS) was used as a novel high-throughput platform in metabolomics^22^ for classification of plasma metabolites modulated by glucose loading and/or training status. Multiplexed separations based on MSI-CE-MS also provide an accelerated workflow for biomarker discovery since time-dependent changes in plasma metabolism are captured within a single run for an individual subject (*i.e.*, personalized metabolomics), including a quality control. The main objective of this work was to identify plasma metabolite signatures that predict glucose tolerance changes after HIIT despite the wide disparity in baseline aerobic fitness and adiposity among sedentary yet normoglycemic women.

## Results

### Cardiometabolic responses of sedentary women to exercise training

Eleven female participants completed all trials in order to evaluate adaptive changes in glucose tolerance associated with exercise training, including two standardized OGTTs performed while fasting before and after a 6 week HIIT intervention. Differential metabolomic responses to HIIT were analyzed by MSI-CE-MS, which were compared with physiological outcomes measured for individual subjects as summarized in Table 1, including changes in aerobic endurance capacity and glucose homeostasis/tolerance. Due to the cross-over design, both within-subject and between-subject changes in maximal oxygen uptake (*VO_2max_*), maximal workload (*W_max_)*, fasting glucose (FG), 2 h post glucose loading (2hPG) and total area under the plasma glucose curve (tAUC) responses were measured with mean/standard deviation for paired subjects of (4.7 ± 3.4) mL/kg/min, (26 ± 15) W, (−0.24 ± 0.92) mM, (−0.063 ± 1.3) mM and (−8.7 ± 161) mM [over 2 h], respectively. Despite consistent positive gains in cardiorespiratory fitness after HIIT in terms of *VO_2max_* (*p* = 1.04 E-3 for paired t-test/two-tailed *n* = 11) and *W_max_* (*p* = 2.35 E-3 for paired t-test/two-tailed *n* = 11), overall improvements in glucose homeostasis/tolerance (*p* > 0.05) were not evident due to large between-subject variations. This was primarily due to the unexpectedly poor glucose response for S2 after HIIT, which when excluded from the data as an outlier (*G* = 0.260 > *G_crit_*; *p* = 0.05) results in an significant change in tAUC from baseline (*p* = 0.0267 for paired t-test/two-tailed, *n* = 10) of (−54 ± 64) mM over 2 hr. Also, there was no correlation (*R^2^* < 0.10) associated with subject body composition (*BMI*, *% fat*) and aerobic fitness or glucose response changes with the exception of Δ2hPG and ΔtAUC (*R^2^* = 0.738) as metrics of glucose tolerance. For example, larger gains in *VO_2max_* after HIIT did not necessarily correspond to better glucose tolerance, nor is *BMI* a good indicator of changes in glucose homeostasis. Moreover, FG outcomes did not correlate with 2hPG or tAUC responses with some subjects having significantly lower FG levels, but with unchanged (*e.g.,* S10) or even reduced glucose tolerance (*e.g.,* S6) after exercise training. One subject (*e.g.,* S2) in particular was found to have negative responses to HIIT in terms of adaptive changes in both FG and P2hG relative to baseline measurements before training. This is in agreement with recent studies that have reported variable responses to exercise training, including susceptible individuals denoted as “adverse responders”, where the observed health effect is opposite to that desired^14^.

### Capturing individual metabolomic responses to OGTT

**Figure 1:**
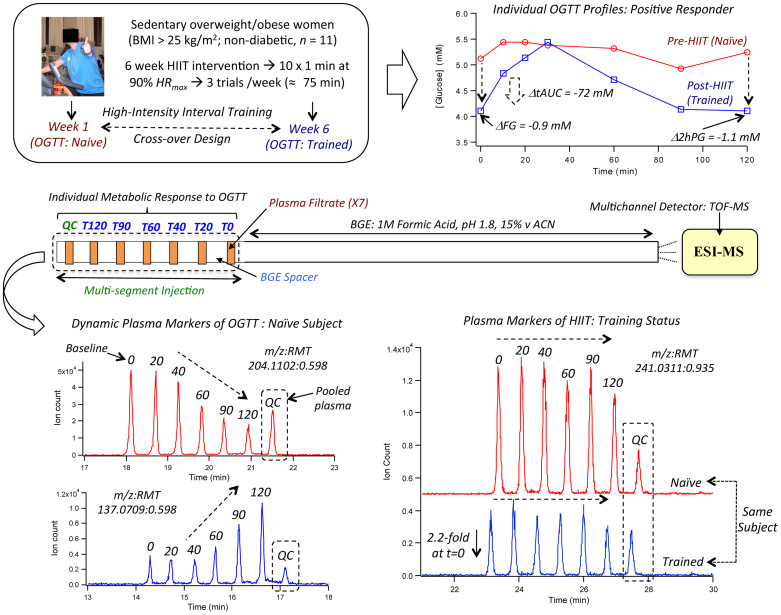
Experimental design and injection configuration used in MSI-CE-MS for elucidating the impact of HIIT on glucose tolerance in sedentary yet normoglycemic women. Dynamic metabolomic responses to oral glucose loading is measured at six time intervals for each subject within a single analysis, including a pooled plasma sample that serves as a quality control. Specific plasma metabolites undergo changes as a function of post-prandial glucose time course and/or training status, which can serve as prognostic markers for predicting outcomes to HIIT intervention, such as changes in oral glucose tolerance (2hPG).

**Figure 2:**
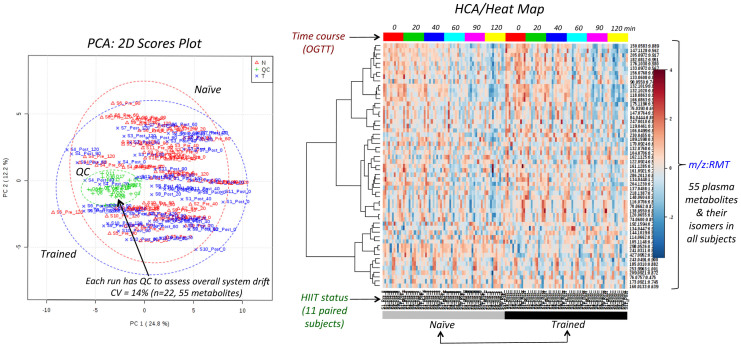
Data overview and quality assurance when using principle component analysis (PCA) that demonstrates good method reproducibility over two days as reflected by the tight clustering of QCs (*n* = 22) in a 2D scores plot relative to biological variance with an average *CV* of 14% for 55 authentic plasma metabolites. Hierarchical cluster analysis (HCA) of auto-scaled and *log*-transformed metabolomic data depicting the data structure of the cross-over study that is dependent on the training status (naïve/trained) and time course (0–120 min) of an oral glucose tolerance test performed on each subject before and after HIIT intervention (OGGT).

MSI-CE-MS was used to explore metabolomic responses to HIIT that underlie variable treatment effects (*i.e.,* glucose tolerance) measured among overweight yet non-diabetic women. Figure 1 depicts the overall study design, including representative OGTT curves for a “positive responder” (*e.g.,* S7) in terms of significant decreases in FG, 2hPG and tAUC outcomes after HIIT intervention. A seven-sample segment serial injection format used in MSI-CE-MS is convenient as it allows for evaluation of metabolomic responses to post-prandial glucose for each subject over six time intervals (relative to baseline) together with a pooled QC in a single run. This enables rapid metabolomic profiling (<5 min/sample) of a large set of plasma filtrates (*n* = 11 × 6 × 2 = 132), including a series of “internal” QCs (*n* = 22) that are used to evaluate system stability. A dilution trend filter was first used as an untargeted primary screen to filter out chemical and biochemical noise using a pooled specimen from the study that enables annotation of authentic plasma molecular features with acceptable reproducibility and linearity^22^. In this way, conventional targeted profiling can be performed on individual plasma specimens when using a validated list of metabolites denoted by their characteristic mass-to-charge-ratio and relative migration time (*m/z*:RMT). The good reproducibility of RMTs (*CV* < 2%) facilitates identification of isomeric and isobaric ions unresolved by MS while also rejecting redundant co-migrating signals during data pre-processing. The same internal standard used for normalization of migration times in CE is also used for correction of changes in injection volume between-samples for reliable metabolite quantification^22^. Figure 1 highlights three distinct metabolomic signal patterns detected by MSI-CE-MS after glucose loading as reflected by a series of extracted ion electropherograms for representative plasma metabolites. Dynamic metabolomic responses associated with post-prandial glucose are readily visualized for plasma metabolites within a single extracted ion electropherogram by MSI-CE-MS. In this case, specific ions from different samples enter the ion source within a narrow time window (≈3–5 min) to allow for unambiguous interpretation of time-dependent changes in circulating metabolism after glucose loading from a fasting baseline condition for individual subjects. Overall, plasma metabolites were found to undergo either a significant decrease or increase in response over time after glucose intake, whereas other metabolites were largely unchanged or they were only modulated after training. Figure 2 illustrates two methods used for assessment of overall data quality, including a 2D scores plot by principal component analysis (PCA) that highlights tight clustering of pooled QCs with an average *CV* ≈ 14% (*n* = 22) over two consecutive days of analysis. The reproducibility of MSI-CE-MS is thus assessed when using a QC incorporated into every separation, which increases sample throughput while ensuring high data fidelity. Also, a hierarchical cluster analysis (HCA) heat map depicts the overall data structure of the study in terms of two factors (*i.e.,* post-prandial glucose time course and training status) involving 11 paired subjects based on 55 unique metabolites denoted by their *m/z:*RMT, which were consistently measured in all plasma samples. The modest number of total plasma metabolites annotated in this work is due to the rigorous data filtering used to reject spurious, background and redundant (*e.g.,* in-source fragments, adducts, isotopes) signals prior to multivariate analysis from 527 total molecular features originally detected in pooled plasma^22^. In addition, only cationic/zwitter-ionic metabolites were measured by MSI-CE-MS under positive ion mode, where concentration sensitivity is limited to low micromolar to sub-micromolar detection limits (*S/N* ≈ 3) due to the small sample volumes (≈10 nL) injected on-capillary^23^.

### Dynamic metabolomic responses to glucose loading and exercise training

**Figure 3:**
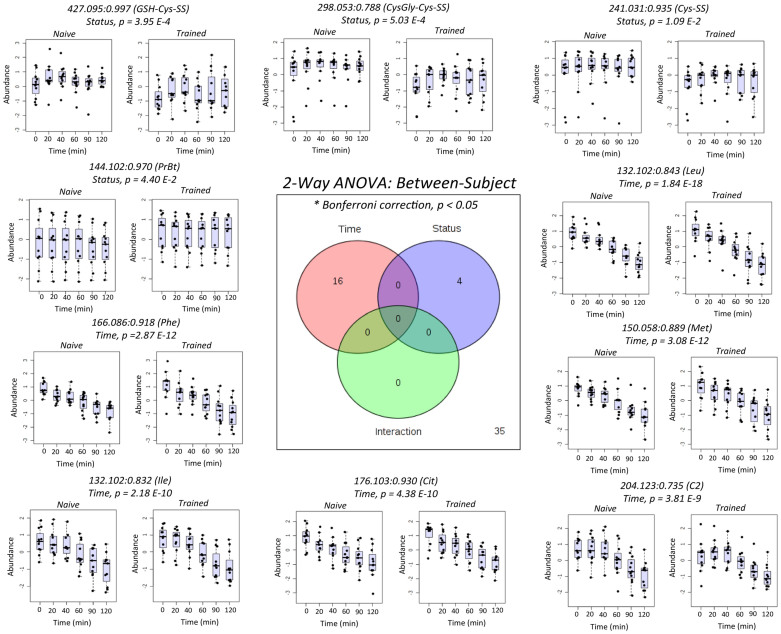
Repeated measures 2-way ANOVA (between-subject) for evaluation of dynamic plasma metabolomic changes associated with time (*i.e.,* post-prandial oral glucose) and/or training status (*i.e.,* HIIT) from 55 authentic plasma metabolites measured by MSI-CE-MS. Overall, plasma oxidized disulfides were lower following exercise training (CysGly-Cys-SS, GSH-Cys-SS and Cys-SS) unlike proline betaine (PrBt), whereas specific amino acids and their intermediates were significantly lower in plasma as depicted by dynamic metabolic profiles after oral glucose loading.

Complementary statistical methods were used to classify plasma metabolites associated with glucose challenge tests and/or training status. Figure 3 summarizes between-subject variance using a repeated measures 2-way ANOVA with Bonferroni correction (*p* < 0.05) to correct for multiple hypothesis testing, where 16 and 4 metabolites were associated with the between-subject variance in post-prandial glucose time course and exercise training, respectively. Metabolites were identified by spiking plasma samples with authentic standards whenever available after searching public databases for putative molecular structures having consistent accurate mass/molecular formula and electromigration behavior (*i.e.,* cations), whereas unknown metabolites remained annotated by their *m/z*:RMT. Oxidized disulfides were the major metabolite class (3 out of 55) significantly modulated after exercise training as reflected by lower circulating levels of cysteinylglycine-cysteine disulfide (GlyCys-Cys-SS), glutathionylcysteine-cysteine disulfide (GSH-Cys-SS) and cystine (Cys-SS). In contrast, the cellular osmolyte tentatively identified as proline betaine (PrBt) was increased in plasma after HIIT training. The majority of other plasma metabolites (39 out of 55) were independent of post-prandial glucose time course. However, several classes of plasma metabolites were found to undergo significant time-dependent decreases after glucose loading (16 out of 55) as summarized in Table 2, including branched-chain (Leu, Ile, Val), aromatic (Phe, Trp, Tyr), sulfur amino acids (Met), as well as urea cycle intermediates (Cit) and carnitine metabolites (C2).

**Figure 4:**
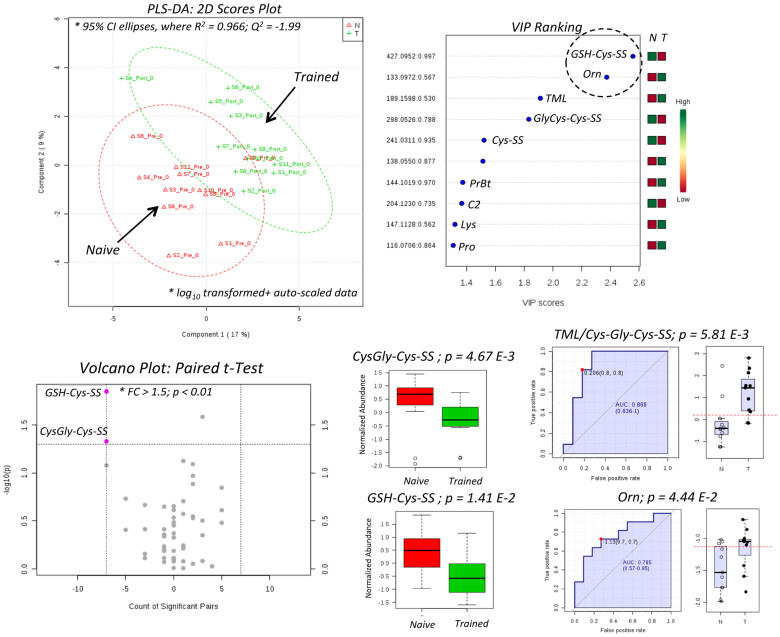
Identification of plasma markers of training status when using PLS-DA, where lower oxidized disulfides (GSH-Cys-SS, GlyCys-Cys-SS, Cys-SS) and higher Orn and TML levels were measured in trained subjects after a 6 week HIIT intervention at rest (0 min). A volcano plot with paired t-test confirms that both GSH-Cys-SS and Orn are significant features (*p* < 0.05) modulated by HIIT. Similarly, receiver operating characteristic (ROC) curves also demonstrates that plasma GlyCys-CysSS and notably TML/GlyCys-Cys-SS ratio are the most significant plasma metabolites for classifying the training status of overweight/obese women.

**Figure 5:**
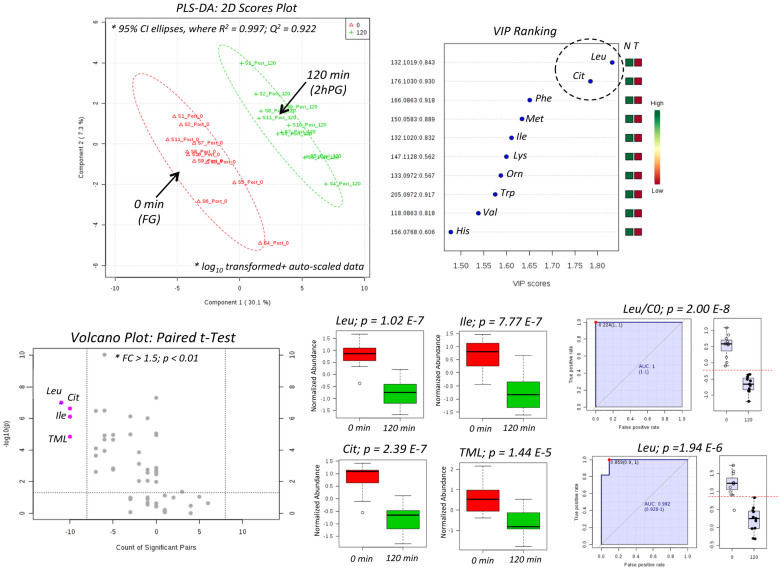
Identification of plasma markers of post-prandial glucose for trained subjects when using PLS-DA, where lower plasma amino acids and their intermediates were measured at 2 h relative to a fasting baseline. A volcano plot with paired t-test confirms that five amino acids and their by-products were most significantly decreased (*p* < 0.001) at 2 h relative to baseline levels. Similarly, receiver operating characteristic (ROC) curves also demonstrates that plasma Leu and notably Leu/C0 ratio are among the most significant plasma metabolites modulated by post-prandial glucose.

Figure 4 confirms that thiol redox status as related to oxidized disulfides (GSH-Cys-SS, GlyCys-CysSS, CysSS) was shifted lower in plasma following HIIT intervention, whereas both Orn and trimethyllysine (TML) were upregulated after exercise training, which comprise the top five candidates in the variable importance in the projection (VIP) when using partial least squares-discriminate analysis (PLS-DA). Although good accuracy was achieved (*R*^2^ = 0.966), the model was not robust based on leave-one-out cross-validation (*Q^2^* < 0). For these reasons other statistical methods were applied to the data matrix, including a volcano plot (fold-change or FC > 1.5; *p* < 0.05) based on a paired t-test that takes advantage of the cross-over study design, which confirms that plasma oxidized disulfides associated with glutathione metabolism were significantly attenuated after HIIT, namely GSH-Cys-SS (*p* = 1.41 E-2) and CysGly-Cys-SS (*p* = 4.67 E-3). Also, receiver operating characteristic (ROC) curves demonstrate good classification of training status based on an overall enhancement in L-ornithine (Orn) status (Orn, *AUC* = 0.785, *p* = 4.44 E-2) that is further improved when using a ratiometric marker (TML/GlyCys-Cys-SS, *AUC* = 0.868, *p* = 5.81 E-3). Figure 5 shows excellent group classification (0 min *vs.* 120 min) after oral glucose loading of trained subjects when using PLS-DA (*R^2^* = 0.997; *Q^2^* = 0.922) as determined by major decreases of plasma amino acids based on the VIP ranking, notably Leu and Cit. Also, a volcano plot with paired t-test highlights that circulating levels of branched-chain amino acids (Leu, Ile), a carnitine precursor (TML) and a urea cycle intermediate, L-citrulline (Cit) were all significantly depleted in plasma 2h after glucose intake (*p* < 2.0 E-4). Furthermore, ROC curves confirm that Leu is the most significant plasma marker of glucose loading after 2 h relative to baseline (*AUC* = 0.992, *p* = 1.94 E-6), whereas the Leu/*L*-carnitine (Leu/C0) ratio provides unambiguous classification of the distinct metabotype (*AUC* = 1.00, *p* = 2.00 E-8) of trained women who transition from a fasting/catabolic to fed/anabolic state. Similar results were found when comparing glucose tolerance responses for untrained women prior to HIIT (data not shown). As expected, glucose challenge tests performed while fasting generate more pronounced changes in plasma metabolism over 2 h relative to adaptive training responses to HIIT that was performed under free-living conditions over 6 weeks and subject to greater biological variance.

### Prognostic plasma markers of glucose tolerance responses to HIIT

**Figure 6:**
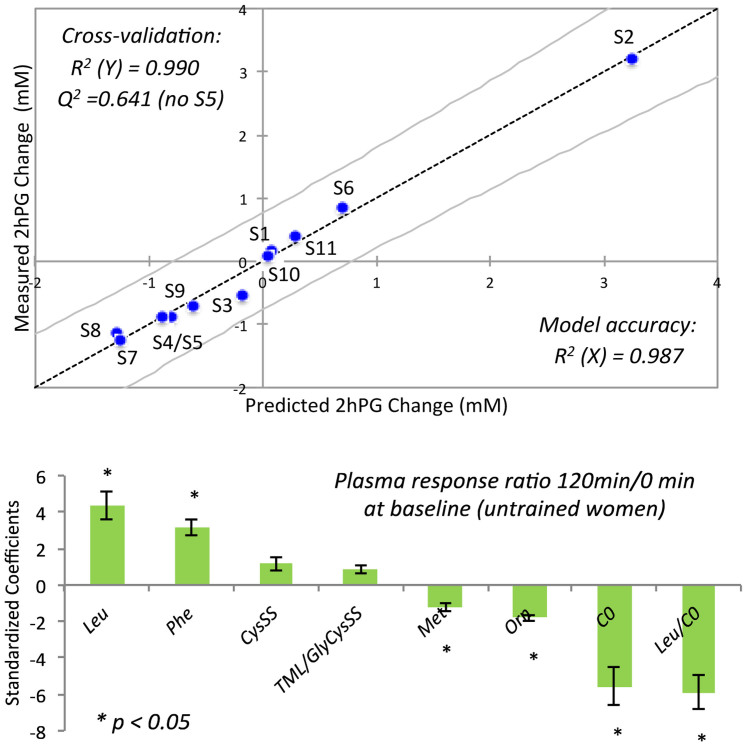
A multiple linear regression (MLR) model (*R^2^* = 0.987 with 95% confidence interval) for predicting changes in 2 h post-glucose (2hPG) response following a 6 week HIIT intervention based on a panel of eight plasma metabolites (six variables with *p* < 0.05) measured at baseline (120 min/0 min) for naïve/untrained women. Good overall model accuracy (*R^2^* = 0.990) and adequate robustness (*Q^2^* = 0.641, S5 was excluded) was evaluated when using leave one-out cross-validation as a permutation test on the original training set. Despite the large between-subject variability, positive responses to HIIT were measured in a major fraction of subjects (54%, 6 out of 11) with significant decreases in 2hPG levels (< 0.50 mM). However, there were also sub-groups of overweight women who had no significant change in 2hPG levels after HIT (27% as non-responders or 3 out of 11) with two participants denoted as negative responders (18% or 2 out of 11) with undesirable glucose tolerance outcomes, notably in the case of S2.

Due to the large between-subject variability in physiological outcomes with HIIT that is uncorrelated with body composition (Table 1), a multiple linear regression (MLR) model was developed to predict changes in glucose tolerance based on a panel of plasma metabolites measured for untrained subjects at 0 min and 120 min that corresponds to the greatest change in baseline metabolism. Metabolites were selected based on their importance as markers of training status (*e.g.,* Cys-SS, TML/GlyCys-Cys-SS, Orn) or glucose loading (*e.g.,* Leu, Leu/C0, Phe) from top candidates identified in PLS-DA, paired t-tests and ROC curves. The MLR model was optimized iteratively by minimizing the number of plasma metabolites to variables while maintaining good accuracy (*R^2^* > 0.900). Permutation testing of the training set was assessed using leave-one-out cross-validation, which demonstrated good model accuracy on average (*R^2^* = 0.990) with adequate robustness (*Q^2^* = 0.641) when predicting changes in 2hPG responses for individual subjects withheld from the original model with the exception of one subject (S5). A similar strategy was also applied for predicting glucose tolerance responses based on changes in tAUC, however the model had lower accuracy (*R^2^* = 0.916) and poor robustness (*Q^2^* = 0.171) after cross-validation. Putative prognostic markers of glucose tolerance included compounds associated with amino acid, thiol redox, urea cycle and carnitine metabolism; however, there was no direct correlation between gains in aerobic endurance capacity and changes in glucose tolerance for trained subjects relative to their naïve state. Figure 6 shows that a panel of eight plasma metabolites as their single or ratiometric variables (six of eight metabolites with *p* < 0.05) predicted changes in 2hPG after a 6 week HIIT intervention with good model accuracy (*R^2^* = 0.987). Although HIIT elicited an improvement in glucose tolerance for a major fraction of the cohort (≈54% or 6 out of 11 subjects with Δ2hPG of 0.5 mM or lower than baseline), there was also a sub-group of non-responders (≈27% or 3 out of 11 subjects with negligible changes in 2hPG) and negative responders (≈18% or 2 out of 11 subjects with Δ2hPG of 0.5 mM or higher than baseline). Overall, Orn status was the most significant variable (*p* = 0.00852) for predicting glucose tolerance changes in sedentary women after exercise training.

## Discussion

In this study, two standardized OGTTs were performed on sedentary women in the morning while fasting prior to and after a 6 week HIIT intervention. Participants in the study were primarily overweight female adults (*BMI* ≈ 27 kg/m^2^ with three women defined as obese *BMI* > 30 kg/m^2^) having a wide disparity in adiposity (≈32–50% body fat) and baseline aerobic fitness (≈19.4–39.3 mL/kg/min), thus representing a heterogeneous cohort. Overall, there were large differences in glucose uptake between-subjects; however, participants did not have impaired fasting glucose levels (*i.e.,* FG < 5.6 mM) or impaired glucose tolerance (*i.e.,* 2hPG < 7.8 mM) with the exception of a subject (S10) who had a slightly elevated FG (6.2 mM) and another subject (S9) with a borderline 2hPG result (7.5 mM) at baseline. Interestingly, in both cases, their glucose responses were restored to within normal ranges after HIIT. The lack of correlation between FG and 2hPG outcomes in this work highlights that these two parameters of abnormal glucose metabolism do not measure the same population while likely underlying different pathophysiological processes in diabetes progression^24^. Overall, a modest improvement in glucose tolerance was only evident when considering changes in tAUC while excluding one subject (S10) who had a notable decrease in glucose tolerance after HIIT intervention. Indeed, subjects with insulin resistance have been reported to derive a greater training-induced change in glucose tolerance than age and *BMI*-matched normoglycemic controls^25^, with men having larger improvements in insulin sensitivity than women^26^. In our case, there was no significant change in weight or insulin sensitivity after short-term HIIT intervention, however modest decreases in abdominal %fat were associated with improvements in insulin area under the curve^12^.

Amino acids were the major class of metabolite that undergo a decrease in plasma over a 2 h time frame after glucose intake that reflects a transition from catabolic (*e.g.,* proteolysis) to anabolic pathways (*e.g.,* protein biosynthesis) as triggered by an oral glucose challenge in a fasting state^20^. Previous studies have shown that insulin levels typically peak around 30 min after glucose loading, whereas plasma amino acids do not return to baseline until after 6 h^27^. Proteolysis is a pathway that is inhibited with glucose loading, resulting in selective tissue uptake of (conditionally) essential amino acids for protein biosynthesis, notably branched-chain (Leu, Ile, Val) and aromatic amino acids (Phe, Trp, Tyr), as well as Met and Lys^28^. This is in agreement with previous metabolomic studies that have noted specific decreases in plasma amino acids after glucose ingestion reflecting reduced protein catabolism due to insulin action^19^,^29^. Leu (*p* = 1.84 E-18) had the most significant time-dependent decrease in plasma among all other polar metabolites, including Ile (*p* = 2.18 E-10) and Val (*p* = 4.90 E-7)^20^,^27^. Also, Phe (*p* = 2.87 E-12) and Met (*p* = 3.08 E-12) were among the top three amino acids (after Leu) that decrease in plasma with post-prandial glucose. Indeed, branched-chain and aromatic amino acids represent predictors of insulin resistance^30^,^31^ as demonstrated recently for circulating levels of Leu, Val and Phe in women after a 6 year follow-up^32^. Similarly, elevated branched-chain amino acids in children is associated with obesity that may independently predict insulin resistance^33^. Thus, the specific composition of dietary amino acids has a profound impact on human health not only as essential macronutrients, but also as modulators of hormonal signalling. For instance, Met restriction extends lifespan while also reducing insulin resistance and body weight gain in mice fed on a high fat diet^34^.

Other plasma metabolites that respond to a glucose challenge include cationic amino acids and their metabolites derived from Lys and Arg. For instance, Orn (*p* = 8.25 E-3) and notably Cit (*p* = 4.38 E-10) are two non-proteinogenic amino acid intermediates of Arg that comprise the urea cycle that decrease with post-prandial glucose. This is in agreement with previous work that has demonstrated that circulating levels of Cit and/or Orn^17^,^19^,^20^,^29^ decrease in response to a glucose challenge. Dysregulation in Arg metabolism is associated with insulin resistance and impaired nitric oxide production in diabetic patients with elevated plasma arginase activity that contributes to vascular aging^35^. Amino acids associated with *L*-carnitine (C0) biosynthesis, including Lys, Met and the non-proteinogenic amino acid, TML, were also found to undergo changes in plasma with post-prandial glucose. C0 is a cofactor for beta-oxidation of fatty acids that mediates their transport as acylcarnitine esters that also prevents deleterious accumulation of acyl-CoA derivatives in the mitochondria. C0 insufficiency due to aging and overnutrition is implicated in insulin resistance and impaired mitochondrial function^36^, whereas C0 therapy has been proposed for the treatment and prevention of diabetes^37^. The ROC curve for Leu/C0 highlights that it is a promising ratiometric marker (*AUC* = 1.00; *p* = 2.00 E-8) for differentiation of glucose loading that may prove useful as a metabolic signature for early detection of diabetes^19^. Acylcarnitines are also associated with post-prandial glucose that indicate a transition from a fasting to a fed state since lipolysis and fatty acid beta-oxidation pathways are inhibited by insulin when glucose serves as the primary source of energy. In our case, *O*-acetyl-*L*-carnitine (C2) was the only acylcarnitine to decrease in plasma over 2 h (*p* = 4.71 E-9) unlike *O*-propionyl-*L*-carnitine (C3)*.* Recent metabolomic studies have shown that plasma C2 serves as a serum marker for predicting impaired glucose tolerance together with glycine and lysophosphotidylcholine^38^. Also, specific medium and long-chain acylcarnitines have been reported to undergo decreases in plasma after glucose loading^39^, which was not measured in this work due to inadequate concentration sensitivity when using MSI-CE-MS^22^. On-line sample preconcentration techniques can further lower detection limits in CE-MS^40^, however it precludes the use of a serial sample segment injection format in multiplexed separations that enhance sample throughput.

One of the major goals of this pilot study is to evaluate the impact of HIIT for enhancing oral glucose tolerance responses on an individual level. To date, there have been few studies examining the efficacy of HIIT to improve body composition and/or insulin sensitivity for overweight yet normoglycemic women as a target population^12^. There are important hormonal factors that contribute to greater biological variability in women (*e.g.,* menstrual cycle) notably when assessing their metabolic responses to an oral glucose challenge^41^. In this work, only 4 out of 55 plasma metabolites were significantly modulated by training status when comparing glucose tolerance responses before and after HIIT when using a repeated measures 2-way ANOVA with Bonferroni correction. Three of the four metabolites represent circulating oxidized thiols as their intact symmetric (Cys-SS) or mixed cysteine disulfides (GlyCys-Cys-SS, GSH-Cys-SS) that decrease from baseline after exercise training. Plasma thiols have previously been reported to be subtly perturbed in response to glucose loading since GSH biosynthesis is inhibited by glucose and activated by insulin^17^. In our case, there was no significant time-dependent change in oxidized disulfides with post-prandial glucose. Cys-SS is the major circulating thiol that plays a key role in regulating the extra-cellular redox state of plasma protein, whereas reduced glutathione (GSH) represents the major intra-cellular antioxidant to maintain redox homeostasis that also mediates detoxification and cell functions. GSH biosynthesis is limited by the availability of Cys that is derived by the influx and reduction of Cys-SS into cells, whereas export of GSH with thiol-disulfide exchange with Cys-SS generates GSH-Cys-SS^42^. Also, recycling of GSH and glutathione *S*-conjugates by γ-glutamyltransferase results in release of GlyCys that forms GlyCys-Cys-SS and other mixed disulfides in plasma. Thus, measurement of plasma thiols provides deeper insight of intra-cellular glutathione homeostasis since exercise training enhances tissue-dependent glutathione antioxidant defenses^43^. The pathological effects of elevated plasma oxidized disulfides has been implicated in diabetes complications due to prolonged hyperglycemia^44^. Physical activity functions as an antioxidant by upregulating antioxidant genes that promote beneficial cell adaptations to exercise-induced oxidative stress^45^. However, exhaustive prolonged exercise without training is deleterious as it can trigger high levels of reactive oxygen species and glutathione depletion that promotes contractile muscle dysfunction and tissue injury. Thus, HIIT provides a sufficient oxidative stimulus via transient bursts of high-intensity exercise followed by periods of recovery/low activity that elicit a low systemic inflammatory response^46^. Overall, the TML/GlyCys-Cys-SS ratio (*AUC* = 0.868, *p* = 5.81 E-3) was found to be the most significant plasma marker for classifying training status using ROC curves. Thus, a reduction of plasma thiol redox status together with upregulation in C0 biosynthetic pathways is associated with positive adaptive metabolic responses to exercise training.

Orn levels were also found to be significantly upregulated after exercise training. Orn is a key substrate in the urea cycle that is required for ammonia detoxification in the mitochondria due to deamination of adenosine monophosphate and branched-chain amino acids as energy sources used by contracting muscle^47^. Indeed, oral supplementation of Orn has been shown to attenuate physical fatigue in subjects performing ergometer cycling by promoting lipid metabolism and mitochondrial function^48^. Overall, Orn was the only plasma metabolite in this study to be modulated by exercise training and oral glucose loading, although the interaction term was not significant. For instance, Orn was among the top ten significant features for classifying P2hG responses from baseline (*AUC* = 0.917, *p* = 1.69 E-4, data not shown), as well as the second most significant plasma marker of training status (*AUC* = 0.785, *p* = 4.44 E-2) when using ROC curves. Proline betaine (PrBt) was the only other plasma metabolite identified by repeated measures 2-way ANOVA to undergo a significant upregulation (*p* = 4.40 E-2) in circulation after HIIT. PrBt is derived from consumption of citrus fruits and it serves as an organic osmolyte in kidney with micromolar concentration levels reported in human plasma^49^. However, due to the lack of an authentic chemical standard, it was only tentatively identified in this work based on its accurate mass (< 5 ppm).

Practical exercise regimes are needed for chronic disease prevention while promoting healthy aging since low cardiorespiratory fitness and physical inactivity are associated with metabolic syndrome^50^. Our work has demonstrated that there is a poor correlation between positive gains in aerobic endurance capacity (*VO_2max_*, *W_max_*) and variable changes in glucose homeostasis (FG) or tolerance (2hPG, tAUC). Recent studies have examined microRNA expression in skeletal muscle to predict differential gains in maximum aerobic capacity with exercise training^51^. Also, plasma metabolites of exercise were found to be correlated with fitness and resting heart rate in healthy individuals reflecting underlying glucose utilization and lipid metabolism^52^. To the best our knowledge, this is the first metabolomics study to model exercise-induced changes in glucose tolerance among overweight yet normoglycemic women. The modest treatment effects are reflected by large between-subject variations in glucose tolerance with Δ2hPG ranging from -1.2 mM (*i.e.,* positive responder, S7) to +3.2 mM (*i.e.,* negative responder, S2) with three subjects (S1, S10 and S11) having non-significant changes (<± 0.4 mM, non-responder). Despite the small sample size and gender-specific cohort examined in this study, the disparity in treatment responses to HIIT as related to Δ2hPG is consistent with large-scale exercise interventions, where ≈ 30% of individuals are reported to have no measurable improvements in insulin sensitivity^26^, whereas ≈ 10% of subjects can have adverse outcomes in terms of risk factors for chronic diseases, including fasting insulin, triglycerides and/or HDL-cholesterol^14^. A MLR model comprising a panel of eight plasma metabolites was developed to predict changes in oral glucose tolerance (Δ2hPG), including key compounds associated with branched-chain/aromatic amino acids, urea cycle, thiol redox status and carnitine metabolism. For instance, Orn (*p* = 0.0085), Phe (*p* = 0.017) and Leu/C0 (*p* = 0.025) were among the most significant prognostic markers associated with Δ2hPG responses measured at baseline. In our work, untrained subjects with attenuated changes in plasma Orn (Δ2hPG) prior to intervention were positively correlated *(r* = 0.613, excluding S5) with improved glucose tolerance outcomes; this was also consistent with the greater training-induced increases to Orn status measured after HIIT intervention. Although there is continued debate on the optimal level of exercise intensity and duration needed to elicit positive health outcomes, integrative lifestyle strategies that include exercise with dietary modifications or modest caloric restriction are needed to improve whole body insulin sensitivity to other peripheral tissues besides contracting skeletal muscle, including adipocytes and liver.

In summary, MSI-CE-MS was used as a high-throughput screening platform for characterizing dynamic metabolomic responses to a glucose challenge on individual subjects both prior to and after a 6-week HIIT intervention. Multiplexed separations greatly enhance the productivity of MS-based infrastructure and data workflow in metabolomics while allowing for unambiguous identification of biomarkers associated with postprandial glucose loading and/or training status that reduces false discoveries. Plasma thiol redox status was reduced following HIIT as reflected by lower circulating levels of oxidized disulfides, whereas exercise-induced upregulation in ornithine status indicated greater intra-cellular antioxidant and detoxification capacity for trained subjects, respectively. Also, specific plasma metabolites were associated with time-dependent peripheral tissue uptake following glucose loading, notably branched-chain/aromatic/sulfur amino acids, as well as urea cycle and carnitine intermediates. Leu/C0 ratio was the most significant plasma marker of glucose loading reflecting a metabolic transition from a fasting to fed state. Also, a panel of plasma metabolites was examined as prognostic markers for predicting variable changes in glucose tolerance (2hPG) among sedentary women following HIIT based on their baseline OGTT. Key limitations of this study include the modest cohort size comprising a single gender that did not examine training effects over a longer time period with a non-exercise control group. In the absence of substantial weight loss, regular exercise is needed to sustain glucose tolerance benefits 72 h after the last exercise bout^26^. In our work, follow-up OGTTs were performed within 72 h of the last HIIT trial. However, the timing of OGTTs did not take into account the menstrual phase of female subjects, which can impact insulin sensitivity contributing to greater metabolic and glucose tolerance variability^53^. A further confounding factor is that HIIT was performed under free-living conditions without explicit dietary control during non-trial dates. Indeed, a single day of dietary standardization has been shown to provide adequate normalization of the human metabolome as measured by NMR with biological variance largely determined by genetics, lifestyle and age/gender^54^. In this study, metabolomic studies using MSI-CE-MS was applied to only cationic/zwitter-ionic metabolites, which excluded the analysis of organic acids, lipids and other anionic metabolites in plasma that are important in understanding gut microbial contributions to exercise and weight loss interventions in obese insulin-resistant women^21^. Also, pre-column chemical derivatization is required to accurately measure plasma thiol redox status in metabolomics that prevents oxidation artefacts while boosting concentration sensitivity for low abundance yet labile reduced thiols^55^. Large-scale studies are needed to validate prognostic markers of exercise-induced glucose tolerance changes as a way to screen for potential adverse responders during baseline testing and customize optimal lifestyle interventions that maximize the salutary benefits of exercise on an individual level.

## Methods

### Study design and cohort selection

Eleven overweight/obese yet healthy women (age: 18–45 years, *BMI*: 25–35 kg/m^2^) participated in the 6-week supervised exercise intervention as previously described. The subjects represent a subset of a group of 16 individuals who took part in a study that evaluated the impact of pre-exercise nutritional state on adaptations to HIIT^12^. Dynamic metabolomic studies were performed using blood samples collected at all six time intervals during two standardized OGTT, which were obtained from only 11 (*i.e.,* 6 fed and 5 fasted) of the 16 original study participants. A placebo arm was not included in this study due to a large body of work demonstrating the efficacy of HIIT to elicit cardiorespiratory fitness gains among sedentary participants^6^,^7^,^8^,^9^,^10^,^11^,^12^. The focus of this study was on evaluating differential treatment responses to HIIT using a repeated measures/cross-over study design, where each subject serves as their own control with a baseline OGTT performed prior to the start of exercise training. Participants were recruited through poster advertisement and the study protocol was carried out in accordance with the approved guidelines of the McMaster University Research Ethics Board. Subjects were classified as “sedentary” based on self-reporting of physical activity ≤ 2 sessions/week of exercise with a duration ≤ 30 min. Subjects were classified as “sedentary” based on self-reporting of physical activity ≤ 2 sessions/week of exercise with a duration ≤ 30 min. Participants completed a general health questionnaire to assess overall health and verify that they did not meet any of the exclusion criteria, including pre-diabetes, diabetes, hypertension or cardiovascular diseases. Informed consent was obtained from all subjects prior to participating in the supervised HIIT intervention trial.

### Body composition, fitness testing and exercise intervention

Body composition analysis to determine percentages of bone, fat, and lean muscle tissue were measured by dual x-ray absorptiometry (DXA) scanning (Lunar Prodigy Advance, Madison, WI). Maximal oxygen uptake (*VO_2max_*), maximal workload (*W_max_*), and average heart rate (*HR*) during exercise trials were determined for each participant using standardized protocols^56^. All of these parameters were measured prior to the start of the exercise training, as well as following the 6-week HIIT intervention for each subject in their naïve and trained states, respectively as previously described^12^. Briefly, exercise was performed on a stationary cycle ergometer (LifeCycle C1, Life Fitness, Schiller Park, IL) while maintaining a pedal cadence of 80–100 rpm. The exercise intervention consisted of 18 HIIT sessions, performed three times a week (with a 1-2 day recovery in between) over a total of 6 weeks. The HIIT protocol involved intermittent cycling and consisted of 10 cycles of 60 s duration at an individualized workload designed to elicit 90% maximal *HR*. Recovery intervals that involved light cycling at 50 W were performed for 1 min between high-intensity bursts for a total exercise time of 25 min for each HIIT trial, which also included a 3 min warm-up and a 2 min cool down period. Standardized breakfasts were only provided for days when the subjects performed HIIT training. All other meals as well as breakfasts on non-training days were under free-living conditions. However, subjects were instructed to maintain their pre-training dietary habits throughout the intervention, which was confirmed by dietary records. The nutritional status of participants during HITT trials was found not to significantly impact exercise training-induced changes in any measured variable including body composition, cardiorespiratory fitness or skeletal muscle remodeling^12^.

### Oral glucose tolerance tests

Subjects underwent an OGTT prior to the start of exercise training, as well as 72 h after the completion of the 6-week HIIT intervention. Subjects fasted for ≥10 h overnight prior to the morning test. An indwelling catheter was inserted into the forearm vein and a resting blood sample was collected and stored on ice before processing. Participants ingested a Glucodex drink containing 75 g glucose and five more blood samples were collected at 0, 20, 30, 60, 90 and 120 min after ingestion. Fasting glucose (FG, 0 min) and 2-h post-glucose loading (2hPG, 120 min) were two key time intervals reflecting glucose homeostasis and glucose tolerance responses, respectively. Vacutainers (6 mL) with EDTA (10.8 mg) as anticoagulant were used for blood collection (Becton Dickinson, Franklin Lakes, NJ, USA). Immediately after collection, blood samples were placed on ice and subsequently centrifuged at 2,500 *g* at 4°C for 5 min to fractionate plasma from blood cells, which were then subsequently frozen at −80°C until analysis. Frozen plasma aliquots were thawed slowly on ice, then vortexed for 30 s to mix gently. Plasma was diluted 4-fold with 200 mM ammonium acetate (pH 5) with 25 μM 3-chloro-L-tyrosine (Cl-Tyr) as internal standard. Plasma proteins were then filtered by ultrafiltration using a 3 kDa MWCO Nanosep centrifugal device (Pall Life Sciences, Washington, NY, USA) at 13,000 *g* for 15 min. A 20 µL aliquot of the diluted plasma filtrate was used for analysis by MSI-CE-MS. Plasma glucose concentrations were determined using a commercial colorimetric enzyme assay kit (Pointe Scientific, Canton, MI, USA), where total area under the glucose curve (tAUC) was integrated using the trapezoidal rule.

### Chemicals and reagents

Ammonium acetate, acetic acid, formic acid, Cl-Tyr and all other metabolite standards were purchased from Sigma-Aldrich (St. Louis, MO, USA). A 10 mM stock solution of Cl-Tyr was prepared in water and stored at 4°C. HPLC-grade acetonitrile (Honeywell, Muskegon, MI, USA) and methanol (Caledon, Georgetown, ON, Canada) were used for preparation of background electrolyte (BGE) and sheath liquid, respectively. All aqueous buffers and stock solutions were prepared with deionized water purified using a Thermo Scientific Barnstead EasyPure II LF ultrapure water system (Cole Parmer, Vernon Hills, IL, USA).

### Instrumentation and method configuration

All CE-TOF-MS experiments were performed using an Agilent G7100A CE system (Mississauga, ON, Canada) interfaced with a coaxial sheath liquid Jet Stream electrospray ion source with heated gas to an Agilent 6230 time-of-flight mass spectrometer (TOF-MS). Nitrogen gas was used as the nebulizer gas in the ESI source and as the drying gas in the MS. An uncoated fused silica capillary (Polymicro Technologies, AZ, USA) with 50 μm ID and 110 cm length maintained at 25°C was used for all experiments. The background electrolyte (BGE) was 1 M formic acid containing 15% *v* acetonitrile (pH 1.8). The applied voltage was 30 kV with a total analysis time of 35 min. In MSI-CE-MS, samples and spacers containing BGE were alternately injected hydrodynamically at 100 mbar as described previously^22^, namely (1) 5 s sample, 40 s spacer; (2) 5 s sample, 40 s spacer; (3) 5 s sample, 40 s spacer; (4) 5 s sample, 40 s spacer; (5) 5 s sample, 40 s spacer; (6) 5 s sample, 40 s spacer; (7) 5 s sample, 5 s spacer. The total injection time for sample and BGE spacer segments was 280 s that is equivalent to about 30% of the total capillary length. Plasma filtrate samples were diluted 4-fold in 200 mM NH4Ac (pH 5) with 25 µM Cl-Tyr as internal standard, which was used for determination of relative migration time (RMT) and relative peak response of metabolites. Between runs the capillary was flushed for 10 min with BGE. The sheath liquid was 60:40 MeOH:H_2_O containing 0.1% formic acid at a flow rate of 10 µL/min via 100:1 splitter. Purine and hexakis(2,2,3,3-tetrafluoropropoxy)phosphazine (HP-921) were spiked into the sheath liquid at a concentration of 0.02% v to produce corresponding reference ions at *m/z* 121.05087 and *m/z* 922.00978 for real time internal mass correction. The TOF-MS was operated in positive-ion mode for detection of cationic/zwitter-ionic metabolites over a range of *m/z* 50-1700 at an acquisition rate of 2 Hz and acquisition time of 500 ms with fixed voltage settings for following: fragmentor = 120 V, skimmer = 65 V and Oct 1 RF = 750 V. In the ESI source the settings were Vcap = 2000 V, nozzle voltage = 2000 V, nebulizer gas = 10 psi, sheath gas = 3.5 L/min at 195°C, and drying gas = 8 L/min at 300°C.

### Sample injection configuration and data workflow

A seven-segment injection format was used in MSI-CE-MS for all analyses in order to enhance sample throughput without loss of information content due to ion suppression^22^. Different injection configurations can be used in order to encode information temporally according to the experimental design, however two specific formats were used in this work. First, a dilution trend filter based on a serial injection of a pooled plasma filtrate sample as quality control (QC) was performed at different dilutions, including a blank (*i.e.,* buffer filtrate)^22^. The QC was prepared by pooling together equal volume aliquots of all individual plasma samples in order to assess system stability, which was also applied in the dilution trend filter in order to annotate common plasma metabolites. Untargeted feature picking was performed using Molecular Feature Extractor (MFE, MassHunter Qualitative Analysis, Agilent Technologies Inc.) in order to detect reproducible yet authentic ions (*i.e.,* [M+H]+ or [M+Na]+) with peak heights over 300 counts while excluding chemical and biochemical noise. MFE was conducted over a time range (≈8–30 min) of the separation while excluding the salt front and the electroosmotic flow (EOF) regions of the separation, where ion co-migration and signal suppression are prevalent. An authentic plasma metabolite from the dilution trend filter was annotated by its characteristic *m/z*:RMT provided the signal was detected with adequate precision (*CV* < 40%, *n* = 3) and linearity (*R^2^* > 0.90) in at least three dilution levels with no signal in the blank sample. This primary screen was initially applied to derive a compound list of authentic molecular features in plasma while filtering spurious signals, background species and redundant ions (*i.e.,* in-source fragments, adduct ions) that comprise the majority of ion signals detected in ESI-MS^22^. A second injection configuration was also applied in MSI-CE-MS when analyzing dynamic metabolome responses to an OGTT at six time intervals (0, 20, 30, 60, 90, 120 min) for each subject either in their naïve or trained state, including a pooled plasma QC. Thus, the same metabolite from seven different samples migrate into the ion source over a short time interval (≈3–5 min) under stable ionization conditions with good quantitative performance. In this way, unambiguous assignment of plasma metabolites associated with glucose loading was realized using an accelerated metabolomics workflow since it allows for targeted analysis of authentic yet reproducible metabolites pre-screened from a dilution trend filter when processing individual plasma samples^22^.

### Quality assurance and statistical analysis

Quality assurance was achieved by inclusion of a pooled QC sample as the seventh sample segment injected during each run performed by MSI-CE-MS (*n* = 22). The QC serves as an internal reference sample included within every separation in order to monitor long-term instrument bias and system drift that is similar to intermittent QCs used in large-scale metabolomic workflows. Moreover, plasma metabolites that were initially identified and validated in the dilution trend filter were only included into the final data matrix if they were detected in a minimum of 75% of all samples with acceptable precision (*CV* < 40%). The latter cut-off limit for precision was typically associated for ions with signals below the quantification limit (*S/N* ≈ 10). This rigorous approach to a metabolomics workflow results in higher data fidelity for biomarker discovery while reducing bias caused by data over-fitting. Multivariate analyses of log-transformed and auto-scaled plasma metabolome data were performed using MetaboAnalyst 2.0^57^, including volcano plot with paired student's t-tests, principal component analysis (PCA), hierarchical cluster analysis (HCA) and partial least-squares-discriminant analysis (PLS-DA). In addition, repeated measures two-way ANOVA with Bonferroni correction was performed to classify plasma metabolites associated with training status and post-prandial glucose response over a 2 h time frame. Receiver operating characteristic (ROC) curves were also performed using ROC Curve Explorer & Tester (ROCCET)^58^ for identification of single or ratiometric (*log*-transformed data) plasma metabolites associated with training status (naïve; trained) and post-prandial glucose (0; 120 min). Multiple linear regression (MLR), as well as normality (Shapiro-Wilk) and outlier (Grubb's) statistical tests for data were performed using Excel (Microsoft Inc., Redmond, WA) with XL STAT (Addinsoft Inc., New York, USA). MLR was used for predicting changes in glucose tolerance (2hPG or tAUC) for individual subjects after HIIT training, which was based on a panel of plasma metabolites measured for naïve subjects at baseline (0 min) and 120 min during the initial OGTT. Plasma metabolites were first selected as putative candidates based on their performance in PLS-DA, paired t-tests and ROC curves, whereas model refining was performed to assess the optimum number of metabolites to retain in the training set by a stepwise regression process in order to maximize predictive accuracy (*R^2^*) and robustness (*Q^2^*) when using leave-one-out at a time cross-validation. All data processing involving electropherograms was performed using Igor Pro 5.0 (Wavemetrics Inc., Lake Oswego, OR).

## Author Contributions

Study conception and design: M.J.G., J.B.G. and P.B.M. Biological sample collection from study participants: M.J.G., J.B.G. and N.L.K. Data acquisition, analysis, and interpretation: P.B.M. and N.L.K. Statistical analysis: P.B.M. and N.L.K. The manuscript was drafted by P.B.M. and N.L.K. Critical revision for important intellectual content: P.B.M., N.L.K. and M.J.G.

## Figures and Tables

**Table 1:** The salutary benefits of a 6 week HIIT intervention involving a cohort of overweight/obese women. Differential responses to exercise training are reflected by changes (in brackets) in aerobic endurance capacity and glucose metabolism from baseline measurements for untrained subjects before intervention

Subject	Age (yrs)	BMI (kg/m^2^)	Fat (%)	VO_2 _max (mL/kg/min)	Workload max (W)	FG (mM)	2hPG (mM)	tAUC (mM, 2h)
S1	24	25.5	33.8	39.3 (−0.90)	248 (+18)	3.8 (+1.1)	4.8 (+0.17)	713 (+85.8)
S2	25	26.3	40.0	31.1 (+4.4)	195 (+30)	5.1 (+0.88)	5.1 (+3.20)	813 (+443)
S3	23	25.7	31.7	26.8 (+9.2)	266 (+20)	5.0 (−0.33)	4.1 (−0.54)	631 (−83.1)
S4	41	27.8	40.1	29.3 (+3.1)	205 (+41)	5.0 (−0.27)	5.5 (−0.88)	734 (−43.1)
S5	23	27.4	47.1	29.8 (+11)	173 (+54)	4.2 (+0.09)	5.9 (−0.88)	784 (−117)
S6	33	25.6	35.1	33.9 (+4.8)	228 (+17)	5.4 (−1.80)	4.2 (+0.86)	507 (−0.77)
S7	41	36.1	50.1	19.4 (+3.0)	190 (+40)	5.1 (−1.00)	5.2 (−1.10)	628 (−71.5)
S8	21	25.1	37.1	30.4 (+7.6)	194 (+12)	4.2 (−0.17)	4.9 (−1.20)	702 (−34.2)
S9	22	27.5	46.6	33.5 (+1.1)	200 (+0)	4.0 (+0.68)	7.5 (−0.71)	950 (−93.9)
S10	20	32.8	48.2	21.2 (+3.8)	166 (+20)	6.2 (−1.50)	6.3 (+0.09)	931 (−144)
S11	23	30.0	50.5	24.1 (+5.3)	170 (+30)	4.0 (−0.28)	4.2 (+0.39)	656 (−33.6)
*Mean*	*27*	*28.2*	*41.8*	*29.0 (+4.7)*	*203 (+26)*	*4.73 (*−*0.24)*	*5.25 (*−*0.063)*	*732 (*−*8.66)*

**Table 2:** Summary of 20 significant plasma metabolites associated with post-prandial oral glucose time course and HIT training status as classified by 2-way ANOVA with Bonferroni correction (*p* < 0.05)

Plasma Metabolite	m/z:RMT	2-Way ANOVA (p-value)	Significance (Metabolic Pathway)
*L*-Leucine (Leu)	132.102: 0.843	1.84 E-18	Branched-chain amino acid; decrease with time
*L*-Phenylalanine (Phe)	166.086:0.918	2.87 E-12	Aromatic amino acid; decrease with time
*L*-Methionine (Met)	150.058:0.889	3.08 E-12	Sulfur amino acid; decrease with time
*L*-Isoleucine (Ile)	132.102:0.832	2.18 E-10	Branched-chain amino acid; decrease with time
*L*-Citrulline (Cit)	176.103:0.930	4.38 E-10	Urea cycle intermediate; decrease with time
*O*-Acetyl-*L*-carnitine (C2)	204.123:0.735	3.81 E-9	Antioxidant/fatty acid oxidation; decrease with time
*L*-Valine (Val)	118.086:0.818	3.90 E-7	Branched-chain amino acid; decrease with time
*L*-Lysine (Lys)	147.113:0.562	4.73 E-6	Cationic amino acid; decrease with time
*N^6^*-Trimethyl-*L*-lysine (TML)	189.160:0.586	5.47 E-5	C0 precursor/fatty acid oxidation; increase with time
*L*-Tryptophan (Trp)	205.097:0.917	9.00 E-5	Aromatic amino acid; decrease with time
C_7_H_16_N_2_O_2_	161.128:0.722	1.81 E-4	Unknown; decrease with time
*L*-Tyrosine (Tyr)	182.081:0.951	1.89 E-4	Aromatic amino acid; decrease with time
*L*-Histidine (His)	156.077:0.606	3.37 E-4	Cationic amino acid; decrease with time
*L*-Ornithine (Orn)	133.097:0.567	8.25 E-3	Urea cycle intermediate; decrease with time and increase with training
Glutathione-cysteine disulfide (GSH-Cys-SS)	427.095:0.997	3.95 E-3	Antioxidant/thiol redox status; decrease with training
Cystinylglycine-cysteine disulfide (CysGly-CysSS)	298.053:0.788	5.03 E-4	Antioxidant/thiol redox status; decrease with training
*L*-Cystine (Cys-SS)	241.031:0.935	1.09 E-2	Antioxidant/thiol redox status; decrease with training
*L*-Arginine	175.119:0.579	2.78 E-2	Cationic amino acid/urea cycle/NO intermediate; decrease with time
*L*-Aspartic acid (Asp)	134.045:1.333	4.17 E-2	Acidic amino acid; decrease with time
Proline betaine (PrBt)	144.102:0.970	4.40 E-2	Osmolyte; decrease with time; increase with training
